# Practice variation among home care nurses

**DOI:** 10.1017/S1463423619000707

**Published:** 2019-10-01

**Authors:** Anne E.M. Brabers, Kim de Groot, Peter P. Groenewegen

**Affiliations:** 1 Nivel – Netherlands Institute for Health Services Research, Utrecht, the Netherlands; 2 Thebe Wijkverpleging (home-care organisation), Tilburg, Noord-Brabant, the Netherlands; 3 Department of Sociology, Department of Human Geography, Utrecht University, Utrecht, the Netherlands

In many European countries, nursing care for patients at home is delivered by home care nurses (Genet *et al*., 2012). Such nursing care varies from personal care (eg, assistance with showering) to technical nursing care (eg, wound care), preventive care (eg, advice to stop smoking) and psychosocial care (eg, support with mourning), both for short and long periods (De Groot *et al*., 2018). In the Netherlands, after a reform of long-term care in 2015 (Kroneman *et al.*, 2016), home care nurses perform the needs assessment for home care. This consists of determining which care is necessary for the individual patient, based on the patient’s care needs, personal situation and social context. No guidelines are available in the Netherlands for performing a needs assessment, although a framework is available including six pre-conditions (eg, specifying the education of nurses who are allowed to do assessments) (V&VN, 2014). An unambiguous and good needs assessment by home care nurses is essential as a starting point for good quality of care. There are, however, signals that home care nurses in the Netherlands assess the care needs for ‘similar’ patients differently (V&VN, 2018). Until now, however, there are only signals that variation in needs assessment exists; there is insufficient insight in the extent of the variation and the underlying causes (V&VN, 2018). For example, a study from 2002, in which care intakers assessed vignettes, already showed practice variation in needs assessment (Buijs *et al*., 2002). A more recent study also indicated variation (Van Dorst *et al*., 2017). In this study, the amount of hours for nursing care assessed by nurses varied from 4 to 13 hours per week for one specific vignette. As possible explanations, the authors hypothesised, but not empirically examined, the culture of the organisation in which the nurses work and their usual practice (Van Dorst *et al*., 2017). The discussion about variation in needs assessments for home care is not unique to the Netherlands. Also in other countries, like England, attention has been paid not only to the different ways but also to the challenges of classifying and exploring needs assessments (Cowley *et al*., 2000).

Variation in needs assessment by home care nurses is not unique; practice variation among physicians has been extensively described in the literature (Paul-Shaheen *et al*., 1987; Wennberg, 2010; Corallo *et al*., 2014). It means that patients with a similar health state or medical condition do not receive the same care. It is often unclear what is behind this variation, as care providers are often not able to clarify why there is variation. For policy makers and funders, variation signals unnecessary care (De Jong, 2008). However, variation is not unambiguous ‘good’ or ‘bad’. The observation that there is variation does not immediately say something about the quality of care. To determine whether variation is warranted or not, insight in underlying causes is necessary. Variation is regarded as warranted if it, for example, can be explained by the patient’s health state or by patient’s legitimate preferences.

Until now, the behaviour of physicians is the focus of most research aimed at getting insight in the underlying causes of practice variation. It showed that factors both at the level of the physician and the environment in which they work influence medical behaviour and therefore variation. Part of the explanations is based on the idea that variation is caused by differences in preferences among physicians for certain treatments. The underlying idea is that physicians apply different treatments because they have learned to value these differently (Wennberg and Gittelsohn, 1975; 1982). Another explanation is one based on a situational argument. The local context of social norms and available capacity influences physician’s behaviour in their daily practice by providing constraints and opportunities (Westert and Groenewegen, 1999). Next to this, recent research showed that patient involvement in medical decision-making influences the decision taken and therefore the variation (Brabers, 2018).

The underlying causes of variation in needs assessment by home care nurses are not known yet. We performed a quick search in PubMed about this topic. Until now, we have not found empirical research that addressed practice variations in home care nursing. Nevertheless, insight in practice variation and its underlying causes are important as it offers directions to improve the needs assessments by home care nurses and in this way reduce unwarranted practice variation. To get insight in the underlying causes, theory-guided research is necessary, based on explanatory mechanisms with associated hypotheses. We hypothesise that causes at the level of the patient, the individual home care nurse, the team in which the home care nurse works, and the organisation where the nurse works have a role in the needs assessment. An example of an explanatory mechanism at the level of the team and the organisation the nurse works in is that of social norms. Care providers adapt to the ‘norm’ within the team or organisation in which they work. They run the risk of being criticised by colleagues who notice the differences in care provision. This risk can be limited by showing the same actions as colleagues (De Jong, 2008). This would lead to the variation in needs assessment within teams or organisations, to be smaller than between teams or organisations. At the level of the home care nurses, it can be reasoned that preferences and previous experiences influence their decisions. At the patient’s level, the social context of the patient may play a role. For instance, it can be argued that less professional care will be needed when the patient has a partner who can support the patient to some extent. The patient’s social context is expected to play a larger role in the clinical choices of home care nurses than in those of physicians. The reason is that needs assessment for nursing care is aimed at strengthening self-management and independence of both the patient and the patient’s social context: what patients can do themselves and what can be expected from their environment is an essential part of the needs assessment (V&VN, 2014). However, an unequivocal assessment of this seems to be more difficult than the assessment of a physical or physiological need.

In sum, in the Netherlands, but in the past also in England, there is a debate about the differences in needs assessment by home care nurses. Such variation is often regarded as unwarranted by policy makers and funders of health care, and as such, they often aim at reducing variation. However, practice variation can be the result of several mechanisms and can also be a sign of patient-centred care. As little is known about this topic among home care nurses, we recommend to first examine the causes of practice variation in needs assessment before policy will be made aimed at reducing practice variation. Only thereafter, it can be determined whether practice variation is warranted or not, and in which way the part that is unwarranted can be reduced. Internationally, there is hardly empirical research to build on. It would be good if the research community takes up the challenges of conducting theory-guided empirical research regarding variation within nursing practice.
